# Identification of a radiosensitivity signature using integrative metaanalysis of published microarray data for NCI-60 cancer cells

**DOI:** 10.1186/1471-2164-13-348

**Published:** 2012-07-30

**Authors:** Han Sang Kim, Sang Cheol Kim, Sun Jeong Kim, Chan Hee Park, Hei-Cheul Jeung, Yong Bae Kim, Joong Bae Ahn, Hyun Cheol Chung, Sun Young Rha

**Affiliations:** 1 Cancer Metastasis Research Center, Yonsei University College of Medicine, Seoul, Korea; 2 Department of Internal Medicine, Yonsei University College of Medicine, Seoul, Korea; 3 Korean Bioinformation Center, Korea Research Institute of Bioscience and Biotechnology, Daejeon, Korea; 4 Department of Radiation Oncology, Yonsei Cancer Center, Seoul, Korea; 5 Brain Korea 21 Project for Medical Science, Yonsei University College of Medicine, Seoul, Korea

**Keywords:** Radiosensitivity, NCI-60, Microarray, Adhesion, Clonogenic assay

## Abstract

**Background:**

In the postgenome era, a prediction of response to treatment could lead to better dose selection for patients in radiotherapy. To identify a radiosensitive gene signature and elucidate related signaling pathways, four different microarray experiments were reanalyzed before radiotherapy.

**Results:**

Radiosensitivity profiling data using clonogenic assay and gene expression profiling data from four published microarray platforms applied to NCI-60 cancer cell panel were used. The survival fraction at 2 Gy (SF2, range from 0 to 1) was calculated as a measure of radiosensitivity and a linear regression model was applied to identify genes or a gene set with a correlation between expression and radiosensitivity (SF2). Radiosensitivity signature genes were identified using significant analysis of microarrays (SAM) and gene set analysis was performed using a global test using linear regression model. Using the radiation-related signaling pathway and identified genes, a genetic network was generated. According to SAM, 31 genes were identified as common to all the microarray platforms and therefore a common radiosensitivity signature. In gene set analysis, functions in the cell cycle, DNA replication, and cell junction, including adherence and gap junctions were related to radiosensitivity. The integrin, VEGF, MAPK, p53, JAK-STAT and Wnt signaling pathways were overrepresented in radiosensitivity. Significant genes including *ACTN1*, *CCND1*, *HCLS1*, *ITGB5*, *PFN2*, *PTPRC*, *RAB13*, and *WAS*, which are adhesion-related molecules that were identified by both SAM and gene set analysis, and showed interaction in the genetic network with the integrin signaling pathway.

**Conclusions:**

Integration of four different microarray experiments and gene selection using gene set analysis discovered possible target genes and pathways relevant to radiosensitivity. Our results suggested that the identified genes are candidates for radiosensitivity biomarkers and that integrin signaling via adhesion molecules could be a target for radiosensitization.

## Background

Predicting tumor response to radiotherapy is one of the major issues in cancer treatment. Predicting radiosensitivity is important for improving clinical outcome and for personalized medicine decisions of the treatment needed, doses, and fractionation schedules [1]. Understanding the mechanism of radiosensitiviy is also a major issue in identifying effective biomarkers and potential drug targets of radiosensitivity [2].

Assays evaluating radiosensitivity have been developed and tested over the last 25 years [3]. Recently, comprehensive gene expression analysis with high-throughput technology has been used to identify radiosensitivity classifiers as well as to elucidate the radiosensitivity mechanism in many cancer types including colorectal, cervical, breast, head and neck cancer [4-7]. As treatment response is related to the complex genetic biology of the cancer and host, biological interaction and factors that determine tumor response through the simultaneous genetic analysis of thousands of genes should be considered in predicting treatment outcome. The cancer cell line panel of the National Cancer Institute (NCI) has been widely used for drug screening based on relevant gene expression [8]. Although promising, these studies are confined to a single platform microarray and further validation and a larger dataset are needed. Moreover, individually identifying every gene with a statistically significant response is not sufficient as a biological explanation. For this reason, gene set analysis is necessary, along with defining the biological processes or pathways in expression analysis.

In this study, to identify a common radiosensitivity gene signature and relevant biological processes from a large amount of data from multiple platforms, we analyzed four types of transcript microarray data from radiosensitivity profiling of the NCI-60 cell line panel. Differentially expressed genes, depending on the radiosensitivity index (survival fraction at 2 Gy of radiation, SF2) were identified using a linear regression model. We hypothesized their roles in radiosensitivity using gene set analysis and pathway analysis.

## Results

### Selection of a common radiosensitivity signature from four microarray platforms

**Figure 1:**
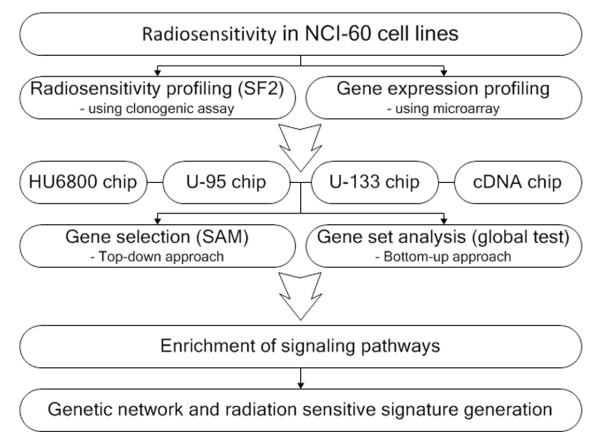
Study scheme of analysis of data from four microarray experiments.

**Figure 2:**
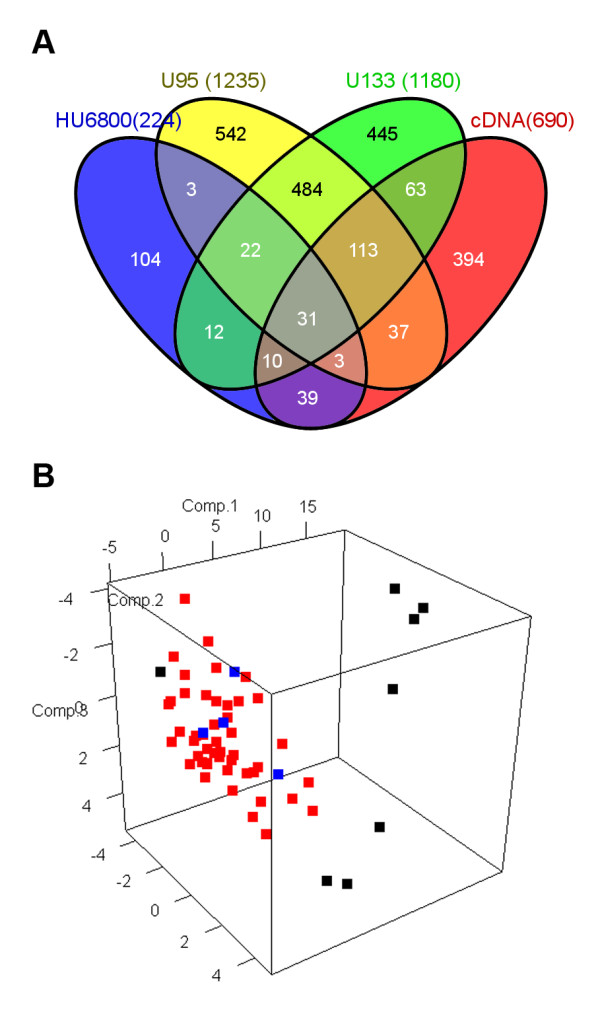
** Identification of 31 radiosensitivity signature genes in NCI-60 cell lines. A**. Venn diagram showing selection of 31 common radiosensitivity signature genes and 179 genes that were selected in more than three platforms from four microarray experiments. **B**. Principal component analysis with gene expression profile using 31 radiosensitivity signature genes. Each cell line is represented as a radiosensitive group (SF2 <0.2; black), an intermediate group (SF2 between 0.2 and 0.8; red), and a radioresistant group (SF2 >0.8; blue).

The study design is in Figure 1. Four published microarray experiments were reanalyzed to identify genes whose expression correlated with radiosensitivity in NCI-60 cancer cell lines. The SF2 radiosensitivity index was determined from previously published literature [9] and considered as a continuous variable ranging from 0 to 1. For gene selection, significant analysis of microarrays (SAM) was applied at the false discovery rate (FDR) of ≤0.10. This resulted in 31 genes commonly identified regardless of platforms and 179 selected from more than three platforms (Figure 2A and Additional file 1). Differences in gene expression between definitely radiosensitive and radioresistant cells by principal component analysis (PCA) showed that approximately the top 10% of radiosensitive (SF2 <0.2) cell lines were distinguished from the bottom 10% of radioresistant lines (SF2 >0.8) using the 31 signature genes (Figure 2B). Of these genes, 21 genes were downregulated and 10 were upregulated in radiosensitive cell lines (Table 1). Reduced expression in a radiosensitive cells meant that decreased gene expression was observed in radiosensitive cells relative to radioresistant cells. Likewise, upregulation meant increased gene expression in radiosensitive cells relative to radioresistant cells. This was determined as the slope of the correlation coefficient between SF2 and gene expression. The scatter plots showing relationships between SF2 and gene expression of the 31 radiosensitivity signature genes in the four microarrays are in Additional files 2,3,4, and 5.

**Table 1:** List of 31 genes selected as a radiosensitivity signature from four microarray platforms

**Symbol**	**Description**	**Location**	**Expression in radiosensitive cell**
ACTN1	actinin, alpha 1	Cytoplasm	Down (0.42)
ANXA2	annexin A2	Plasma Membrane	Down (0.36)
ANXA5	annexin A5	Plasma Membrane	Down (0.42)
ARHGDIB	Rho GDP dissociation inhibitor (GDI) beta	Cytoplasm	Up (−0.49)
CAPNS1	calpain, small subunit 1	Cytoplasm	Down (0.48)
CBR1	carbonyl reductase 1	Cytoplasm	Down (0.41)
CCND1	cyclin D1	Nucleus	Down (0.54)
CD63	CD63 molecule	Plasma Membrane	Down (0.51)
CORO1A	coronin, actin binding protein, 1A	Cytoplasm	Up (−0.46)
CXCR4	chemokine (C-X-C motif) receptor 4	Plasma Membrane	Up (−0.46)
DAG1	dystroglycan 1 (dystrophin-associated glycoprotein 1)	Plasma Membrane	Down (0.60)
EMP2	epithelial membrane protein 2	Plasma Membrane	Down (0.41)
HCLS1	hematopoietic cell-specific Lyn substrate 1	Nucleus	Up (−0.58)
HTRA1	HtrA serine peptidase 1	Extracellular Space	Down (0.52)
ITGB5	integrin, beta 5	Plasma Membrane	Down (0.47)
LAPTM5	lysosomal protein transmembrane 5	Plasma Membrane	Up (−0.50)
LRMP	lymphoid-restricted membrane protein	Cytoplasm	Up (−0.49)
MYB	v-myb myeloblastosis viral oncogene homolog (avian)	Nucleus	Up (−0.59)
PFN2	profilin 2	Cytoplasm	Down (0.61)
PIR	pirin (iron-binding nuclear protein)	Nucleus	Down (0.43)
PKM2	pyruvate kinase, muscle	Cytoplasm	Down (0.44)
PTMS	parathymosin	Nucleus	Down (0.48)
PTPRC	protein tyrosine phosphatase, receptor type, C	Plasma Membrane	Up (−0.55)
PTPRCAP	protein tyrosine phosphatase, receptor type, C-associated protein	Plasma Membrane	Up (−0.49)
PYGB	phosphorylase, glycogen; brain	unknown	Down (0.35)
RAB13	RAB13, member RAS oncogene family	Plasma Membrane	Down (0.43)
RALB	v-ral simian leukemia viral oncogene homolog B (ras related; GTP binding protein)		
		Cytoplasm	Down (0.47)
SCRN1	secernin 1	Cytoplasm	Down (0.40)
SQSTM1	sequestosome 1	Cytoplasm	Down (0.48)
TWF1	twinfilin, actin-binding protein, homolog 1 (Drosophila)	Cytoplasm	Down (0.43)
WAS	Wiskott-Aldrich syndrome (eczema-thrombocytopenia)	Cytoplasm	Up (−0.60)

“Down” refers to decreased expression observed in radiosensitive cells relative to radioresistant cells, determined as the slope of the correlation coefficient. “Up” refers to increased expression in radiosensitive cells.

### Integrative functional gene set analysis using a global test

To explain the biological processes and signaling pathways of radiosensitivity, a gene set functional study using a global test [10] was applied. The selected gene set was defined from the Kyoto encyclopedia of genes and genomes (KEGG) pathways. The adjusted p-value corrected for multiple comparisons using the Benjamini and Hochberg method [11] is in Table 2. Several radiation-related functions were enriched including the cell cycle, DNA replication, cell junction, and cell adhesion (Table 2A). In addition, several molecular pathways were overrepresented including the integrin, vascular endothelial growth factor (*VEGF*), mitogen-activated protein kinase (*MAPK*), *p53*, and *Wnt* signaling pathways (Table 2B).

**Table 2:** Gene set analysis using Kyoto encyclopedia of genes and genomes (KEGG) pathways

**A**					
**Function**	**KEGG pathway**	**cDNA**	**HU-6800**	**U133**	**U95**
Cell cycle & DNA replication					
	Cell cycle	0.019	0.019	< 0.001	< 0.001
	DNA replication	0.001	0.125	0.003	0.001
	Base excision repair	0.003	0.003	0.002	0.003
Cell junction					
	Adherens junction	< 0.001	< 0.001	< 0.001	0.001
	Tight junction	0.004	0.003	0.002	0.006
	Gap junction	0.011	0.007	0.015	0.014
					
Cell adhesion molecules					
	Focal adhesion	0.016	0.315	0.014	0.015
	Cell adhesion molecules	0.007	0.007	0.017	0.005
	Regulation of actin cytoskeleton	0.013	0.013	0.001	< 0.001
**B**		**cDNA**	**HU-6800**	**U133**	**U95**
Molecular pathway					
	Integrin signaling pathway	< 0.001	0.004	0.004	0.001
	VEGF signaling pathway	0.003	< 0.001	< 0.001	< 0.001
	Phosphatidylinositol signaling	0.009	0.009	0.006	0.003
	Wnt signaling pathway	0.004	0.524	< 0.001	< 0.001
	Jak-STAT signaling pathway	0.034	0.034	< 0.001	0.003
	MAPK signaling pathway	0.017	0.017	0.002	0.003
	ErbB signaling pathway	0.014	0.005	0.021	0.014
	p53 signaling pathway	0.035	0.034	0.016	0.006

Adjusted p-value using using Benjamini and Hochberg method as represented in four microarray platforms (significance cutoff is adjusted p-value < 0.01).

### Adhesion-related molecules as major components in a radiosensitivty signature

To integrate the top-down and bottom-up approach, 31 radiosensitivity signature genes found through SAM analysis were compared with the gene sets found in the global test. Eight genes were functionally annotated in the global test, and their major function was examined according to KEGG pathways (Table 3). The common function was related to cell junctions and adhesion, suggesting that adhesion-related molecules might have a major role in the mechanism of radiosensitivity.

**Table 3:** Eight genes encoding adhesion molecules in the radiosensitivity signature and related function in KEGG pathways

**Symbol**	**Entrez Gene Name**	**Related function in KEGG pathways**
CCND1	cyclin D1	Cell Cycle, Focal adhesion, Jak-STAT signaling pathway, p53 pathway, Wnt signaling pathway
ACTN1	actinin, alpha 1	Focal adhesion, Tight junction, Adherens junction, Regulation of actin cytoskeleton
WAS	Wiskott-Aldrich syndrome	Regulation of actin cytoskeleton, Adherens junction,
HCLS1	hematopoietic cell-specific Lyn substrate 1	Tight junction
RAB13	RAB13, member RAS oncogene family	Tight junction
PTPRC	protein tyrosine phosphatase, receptor type, C	Cell adhesion molecules (CAMs)
ITGB5	integrin, beta 5	Focal adhesion, Regulation of actin cytoskeleton
PFN2	profilin 2	Regulation of actin cytoskeleton

### Genetic network interaction with adhesion-related molecules in the integrin signaling pathway

**Figure 3:**
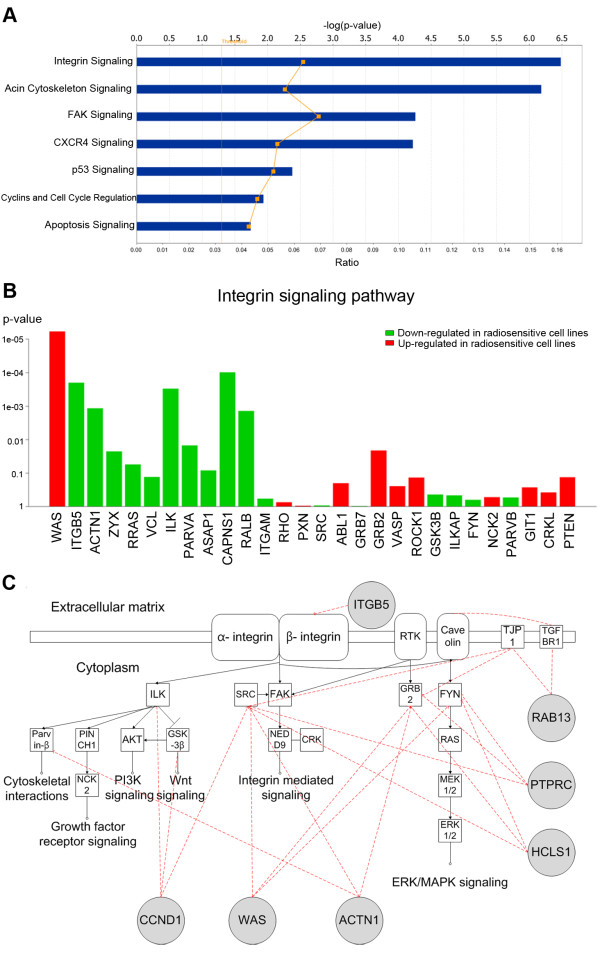
** Integrin signaling pathway and its interaction as a radiosensitive target. A**. Statistical ranking of pathways with the commonly selected 179 genes using SAM analysis. The x-axis displays the -log of the p-value calculated by Fisher's exact test, right-tailed. **B**. Gene plot showing the influence of individual genes of the integrin signaling pathway produced by a global test. The influence on the y-axis is represented as the p-value, the extent of correlation between SF2 (radiosensitivity) and gene expression in a gene set. A lower p-value means that the gene is well correlated between SF2 and the gene expression value. **C**. Integrin signaling pathway interaction with identified adhesion molecules from the 31 radiosensitivity signature. (References from Ingenuity knowledge base, Additional file 6).

To generate a genetic network for radiosensitivity, we performed ontology analysis using 179 genes that were selected from more than three platforms using SAM analysis. Statistical ranking with canonical pathways was performed using ingenuity pathway analysis (IPA) (Figure 3A). Overrepresented pathways were adhesion-related pathways including the integrin, actin-cytoskeleton, and focal adhesion kinase (FAK)-signaling pathway. In addition, the cell cycle and p53 signaling pathways important to radiosensitivity were also identified. To identify the influence of each gene on the integrin signaling pathway, which was the most overrepresented pathway, a gene plot was produced using the gene set determine from the global test (Figure 3B). Among the 31 signature genes, several were enriched, including *ACTN1*, *CAPNS1*, *ITGB5*, *RALB*, which were downregulated, and *WAS*, which was upregulated in radiosensitive cell lines. Genetic network interaction showed that adhesion-related molecules in Table 3 were involved in the integrin-signaling pathway, and that interaction existed with other signaling pathways such as the PI3K, Wnt, and MAPK signaling pathways, which were enriched, as shown in Table 2B (Figure 3C, Additional file 6).

## Discussion

The discovery of potential biomarkers and the elucidation of the mechanisms of radiosensitivity are important to developing radiosensitizers as well for predicting tumor response in radiation oncology [2,12]. We reanalyzed four published microarray studies to identify a common radiosensitivity signature regardless of platform. This strengthened the reliability of our analysis. Using SAM, we examined each gene individually to show that the correlation with SF2 was significant. Next, we performed a gene set analysis using a global test based on a linear regression model with a well-defined gene set from KEGG pathways. A combination of both analyses found that adhesion-related molecules and several cancer-related molecular pathways were significantly enriched for radiosensitivity and these molecules were linked via the integrin signaling pathway. Using both a top-down and bottom-up approach increases the ability to determine genes and signaling pathways that are biologically explainable and statistically acceptable.

Several studies have reported possible radiosensitivity predictive genes [4,7,13,14]. However, no gene is common among the previous reports. Therefore, we used four microarrays to find genes commonly identified as significant in radiosensitivity. We identified 31 common genes as well as 179 genes that were selected in more than three studies (Table 1 and Additional file 1). Of these 179 genes, 8 were previously reported [7,9,15,16] (Additional file 7). Comparing the 179 genes with previous reports, the cell cycle genes *CCNA2* and *CDK6* in esophageal cancer [16], and the ras-related gene *RAC2* in rectal cancer [5] were common. Other genes that were reported previously could also be possible drug targets. The 31 signature genes had cellular functions including cell cycle and DNA repair, cell junction, and cell adhesion. Cyclin D1 (*CCND1*) is well known as a DNA repair gene and might sensitize human cancers to radiation by limiting DNA repair [15]. In breast cancer, overexpression of cyclin D induces radiation resistance by inhibiting apoptosiss [17]. In our analysis, *CCND1* was downregulated in radiosensitive cell lines, consistent with this explanation. Annexins including *ANXA2* and *ANXA5* are family of Ca^2+^-regulated membrane-binding proteins that interact with the cellular membrane. *ANXA5*, in particular, is related to induction of apoptosis and is used as an apoptosis marker [18]. *ACTN1WASHCLS1RAB13*, and *PFN2* are involved with cellular junctions and the actin cytoskeleton, and *PTPRC* is known for interacting with cell adhesion molecules. Cellular adhesion-mediated radioresistance is proposed to generate anti-apoptotic signals when integrin-mediated adhesion interacts with the extracellular matrix (ECM) [19,20].

Integrins are adhesion molecules localized in the plasma membrane, and are heterodimeric glycoprotein receptors of α- and β-subunits. They directly bind to the ECM and contribute to proliferation, survival, and invasion in cancer [21]. In radiation biology, several studies report integrins as prognostic or therapeutic markers in several cancer types including breast, head and neck, prostate, lung, and colon cancer [22,23]. In addition to integrin β1, which was included in our identified 179 genes and the most studied relative to radiosensitivity, our study identified integrin β5 (*ITGB5*) as a radiosensitive gene. αvβ5 receptors are considered to be potential therapeutic targets because of their anti-angiogenic and anti-metastatic effects, and cilengitidem, which is known as αvβ5 antagonist, has been studied in anti-cancer therapy [24]. Likewise, *ITGB5* could be a potential biomarker as a prognostic marker or radiosensitizer in radiotherapy. Using systems biology, we showed that major cancer-related signaling pathways were enriched related to radiosensitivity (Table 2B) and that the integrin signaling pathway interacts with other pathways, including *MAPKWnt*, and *PI3K* signaling, as shown in Figure 3B. These findings suggest that integrin signaling with identified adhesion molecules could be central in radiosensitivity and one of the common radiosensitivity mechanisms, regardless of cell type. Our work could be the basis for future biological validation targeting integrin signaling pathways in radiosensitization.

Although we identified a common radiosensitivity signature regardless of cell type, radiosensitive cells (SF <0.2) included cells of lymphoid origin and could have introduced bias in analysis. To exclude the effect of lymphoid origin, we adjusted correlation coefficients and p-values between radiosensitive cells (SF2 <0.2) and radioresistant cells (SF2 ≥0.2) using mean-centering and a standardization method [25] (Additional file 8). We observed that correlation coefficients of the 31 radiosensitivity signature genes were similar before and after adjustment for the four microarrays. Therefore, we used the microarray data without artificial adjustment for cell type, which could change the true values of the experimental data.

There are two limitations to this study. First, we used NCI-60 cancer cell lines to identify common radiosensitivity signatures regardless of cell type. Defining common radiosensitive mechanisms not affected by cell type is helpful, but the actual cellular response in biological validation might differ among cell types. However, we adjusted for this effect using statistical methods. Adjusted correlation coefficients were similar to correlation coefficients before adjustment. Second, although we identified a gene signature using four microarray array platforms, using not only mRNA expression, but also comparing DNA sequences or protein expression would give a comprehensive analysis of the radiosensitivity mechanism.

## Conclusions

A common radiosensitivity gene signature was identified that involved 31 genes. Their major functions were in the cell cycle, cell junctions, and cell adhesion. Adhesion-related molecules were enriched in the integrin signaling pathway and could be targeted for radiosensitization. This is the first study to use multiple microarray platforms to study radiosensitivity, and might provide insights in elucidating novel therapeutic targets and common radiosensitivity mechanisms regardless of cell type.

## Methods

### Radiosensitivity profiling and mRNA expression profiling

Radiosensitivity profiling was defined as the survival fraction at 2 Gy radiation (SF2) [9]. Radiosensitivity signature genes were identified from previously published SF2 data on radiosensitivity profiling and gene expression profiling [8,9,26,27] of the NCI-60 cell line panel. Briefly, the cells had been plated and radiated with 2 Gy of x-rays. After fixation, colonies of over 50 cells were calculated. SF2 was determined by the formula: (SF2 = number of colonies/total numbers of cells plated × plating efficiency). SF2 ranged from 0 to 1, with a lower SF2 representing more radiosensitivity. Gene expression profiling data using the NCI-60 cancer cell line panel was from cDNA and two-color arrays [8], and HU-6800 [26], HG-U133 [27], and HG-U95 Affymetrix microarrays [27], and obtained from Cellminer (http://discover.nci.nih.gov/cellminer) and http://www.broadinstitute.org/mpr/NCI60/NCI60.html. The gene expression data were acquired from the National Center for Biotechnology Information Gene Expression Omnibus (GPL1290, GSE5720, and GSE5949). Gene annotations were obtained from the SOURCE database (http://smd.stanford.edu/cgi-bin/source/sourceSearch. After gene annotation, we matched gene symbols among the four microarray platforms. For normalization, robust multiarray analysis (RMA) was used for normalizing Affymetrix gene chips and the linear models for microarray data (LIMMA) package was used with the R program for normalizing the two-color array [28]. Missing values were imputed using the K-nearest neighbor method. All acquired microarray data were from experiments using cells in the unirradiated status.

### Identification of a common radiosensitive gene signature

A radiosensitive signature gene was defined as a gene whose mRNA expression correlated with SF2. SF2 was defined as a continuous variable. Gene selection was performed using SAM [29]. Parameters for SAM analysis were test statistic, T-statistic, number of permutations = 1000, false discovery rate (FDR) = 0.1. Correlation between mRNA expression and SF2 was calculated using a quantitative method calculating the linear regression coefficient between gene expression and radiosensitivity from the linear regression model, and genes were identified under a false discovery rate of 10% for all four microarray platforms. We performed SAM for each microarray platform. A common radiosensitive gene signature were defined as genes commonly identified in all four microarray platforms. A Venn diagram was plotted using Venny (http://bioinfogp.cnb.csic.es/tools/venny/index.html). Principal components analysis was performed for data reduction, simplifying datasets to three dimensions for plotting purposes. Principal component analysis was conducted using R statistical software (http://www.r-project.org), using “princomp()” function and default options.

### Gene set enrichment analysis using a global test

To find a pathway of genes correlated with SF2, gene set analysis was performed using a global test [10] with a defined gene set from the Kyoto encyclopedia of genes and genomes (KEGG) pathways. This test was based on the generalized linear model and tested the null hypothesis in which all regression coefficients between SF2 and gene expression were zero. This was a score test based on random-effect modeling of parameters corresponding to the coefficients of the individual genes in a pathway. It was used to determine whether the global expression pattern of a gene set was significantly related to SF2. If the global test was significant, the genes in the gene set were more associated with SF2 than expected under a null hypothesis (not associated with SF2). These associations could involve both upregulation (positive) and downregulation (negative). Typically, a significant gene set is a combination of positively and negatively related genes. P-value was corrected for multiple comparisons using the Benjamini and Hochberg method [11]. An adjusted p-value under 0.01 was considered as significant. Analysis was done using R statistical software. We used function “gt” in the R package “globaltest”.

### Canonical pathway analysis, gene plot and genetic network representation

In addition to gene set analysis, canonical pathway analysis was performed using 179 genes identified in more than three microarray platforms using SAM analysis. Canonical pathway analysis identified the pathways from the Ingenuity Pathways Analysis library of canonical pathways that were most significant to the 179 genes. In this test, the p-value was measured to decide the likelihood that the association between 179 genes and a given pathway was due to random chance. The smaller the p-value, the lower the likelihood of random association and the more significant the association. The significance of association between the 179 genes and the canonical pathways was measured in two ways: 1) the ratio of the number of molecules from the data set that mapped to the pathway divided by the total number of molecules that mapped to the canonical pathway; 2) Fisher’s exact test to calculate a p-value determining the probability that the association between the genes in the data set and the canonical pathway was explained by chance alone. The Benjamini-Hochberg method of multiple testing was used for correction [11].

A gene plot exploits the fact that a global test statistic for a set of alternative covariates can be written as the weighted sum of the global test statistics for each single contributing covariate. Displaying these component global test results in a bar plot that gives insight into the subset of covariates that is most responsible for the significant test result. The plot showed the p-values of the component tests on a reversed log scale. The influence on the y-axis means the extent of correlation between SF2 and gene expression in a gene set. A lower p-value meant that the gene was well correlated between SF2 and the gene expression value.

In the genetic network, molecules were represented as nodes, and the biological relationship between two nodes was represented as an edge. All edges were supported by at least one reference from the literature, a textbook, or canonical information stored in the Ingenuity Pathways Knowledge Base. Data were analyzed using Ingenuity Pathways Analysis (Ingenuity Systems, http://www.ingenuity.com).

## Competing interests

The authors declare that they have no competing interests.

## Authors’ contributions

HSK and SCK performed analysis, interpreted the results and drafted the manuscript. SJK and CHP participated in collection and preprocessing of microarray data. JBA and YBK helped to build the research scheme and data analysis method. HCJ and HCC participated in intellectual discussions. SYR planned this study and supervised the research team. All authors read and approved the final manuscript.

## Supplementary Material

Additional file 1 ** List of 179 genes selected in more than three platforms.** “Down” refers to decreased expression observed in radiosensitive cells relative to radioresistant cells, determined as the slope of the correlation coefficient. False discovery rate was calculated from SAM analysis. Click here for file

Additional file 2 Scatter plots of the 31 radiosensitivity signature genes between gene expression and radiosensitivity (SF2) in cDNA microarray. Click here for file

Additional file 3 Scatter plots of the 31 radiosensitivity signature genes between gene expression and radiosensitivity (SF2) in HU-6800 microarray. Click here for file

Additional file 4 Scatter plots of the 31 radiosensitivity signature genes between gene expression and radiosensitivity (SF2) in Affy U133 microarray. Click here for file

Additional file 5 Scatter plots of the 31 radiosensitivity signature between gene expression and radiosensitivity (SF2) in Affy U95 microarray. Click here for file

Additional file 6 Interaction between identified radiosensitivity genes and target genes for the integrin signaling pathway from the Ingenuity knowledge base. Click here for file

Additional file 7 Comparison with published radiosensitivity signatures. Previously reported genes in our gene lists are highlighted in yellow. Click here for file

Additional file 8 Adjusted correlation coefficient and p-value of 31 radiosensitivity signature genes to remove the effect of lymphoid origin using mean-centering and standardization method in four microarrays. Click here for file
