# Supplementary material for: Identification of a radiosensitivity signature using integrative metaanalysis of published microarray data for NCI-60 cancer cells
Source: BMC Genomics. 2012 Jul 30;13:348. doi: 10.1186/1471-2164-13-348 (PMC3472294; doi:10.1186/1471-2164-13-348)
Supplement: Additional file 2 — Scatter plots of the 31 radiosensitivity signature genes between gene expression and radiosensitivity (SF2) in cDNA microarray. [file 1471-2164-13-348-S2.pdf]

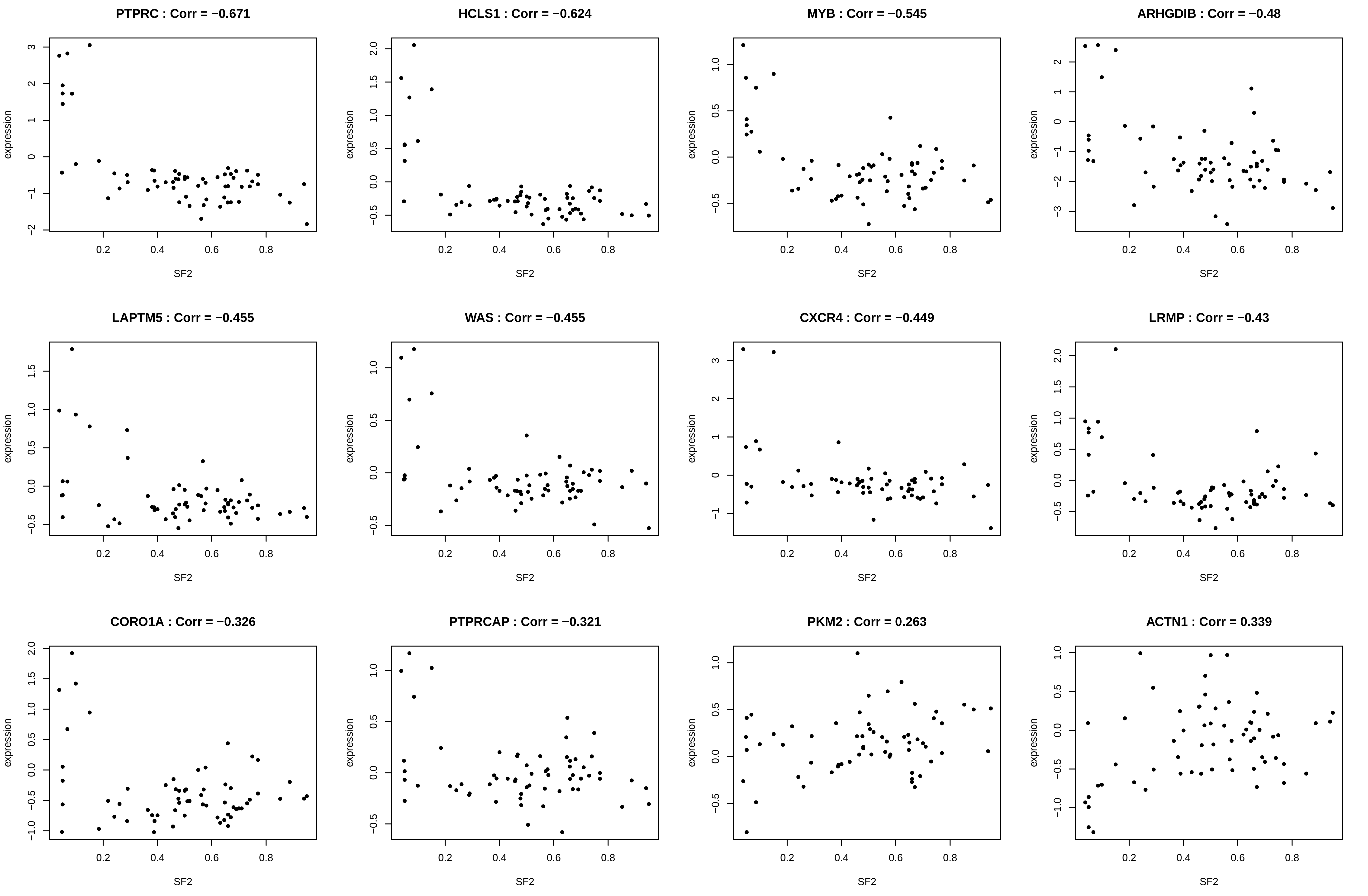
PTPRC : Corr = −0.671
HCLS1 : Corr = −0.624
MYB : Corr = −0.545
ARHGDIB : Corr = −0.48
LAPTM5 : Corr = −0.455
WAS : Corr = −0.455
CXCR4 : Corr = −0.449
LRMP : Corr = −0.43
CORO1A : Corr = −0.326
PTPRCAP : Corr = −0.321
PKM2 : Corr = 0.263
ACTN1 : Corr = 0.339
expression
SF2

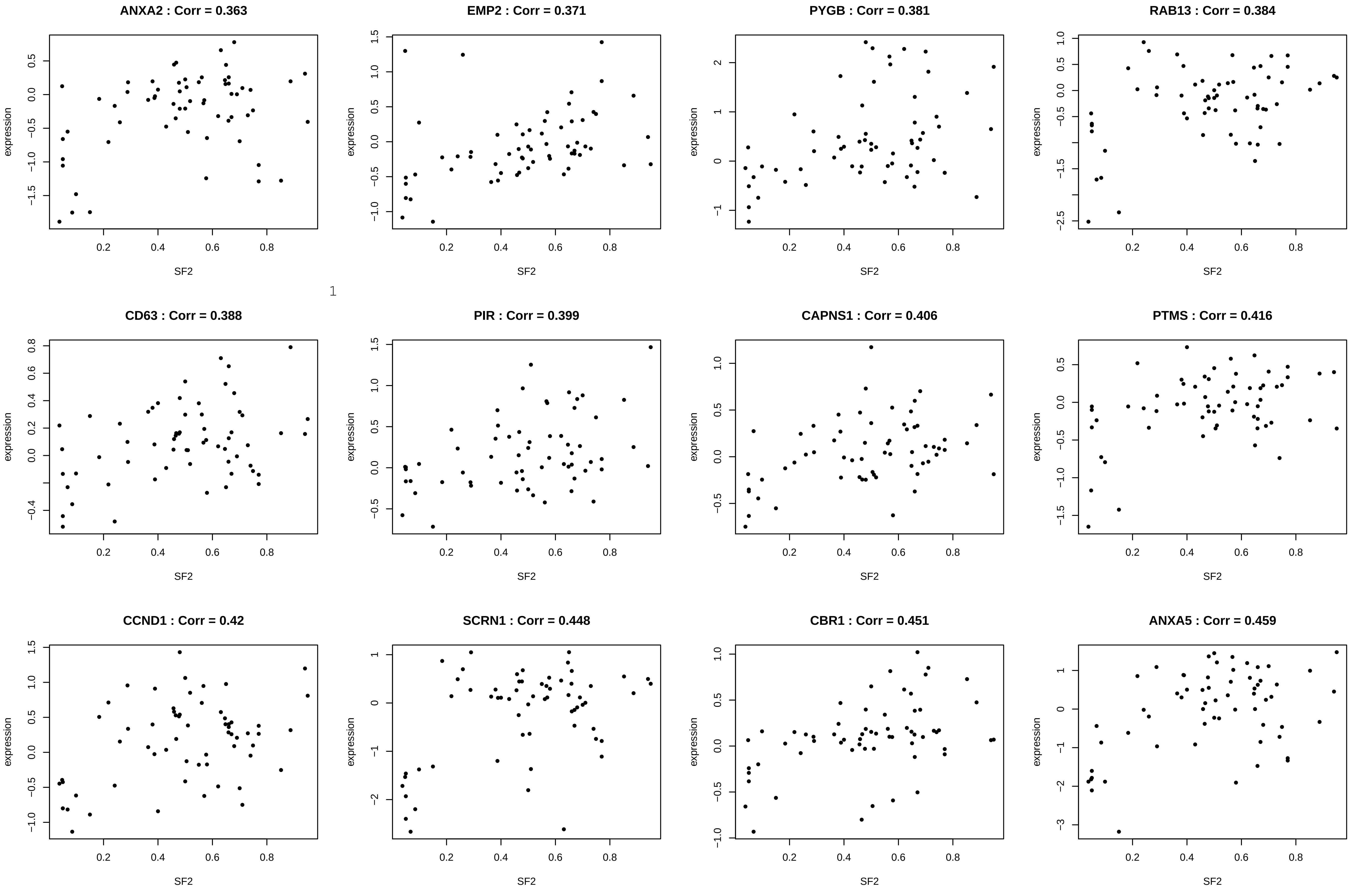

ANXA2 : Corr = 0.363
EMP2 : Corr = 0.371
PYGB : Corr = 0.381
RAB13 : Corr = 0.384
CD63 : Corr = 0.388
PIR : Corr = 0.399
CAPNS1 : Corr = 0.406
PTMS : Corr = 0.416
CCND1 : Corr = 0.42
SCRN1 : Corr = 0.448
CBR1 : Corr = 0.451
ANXA5 : Corr = 0.459
expression
SF2

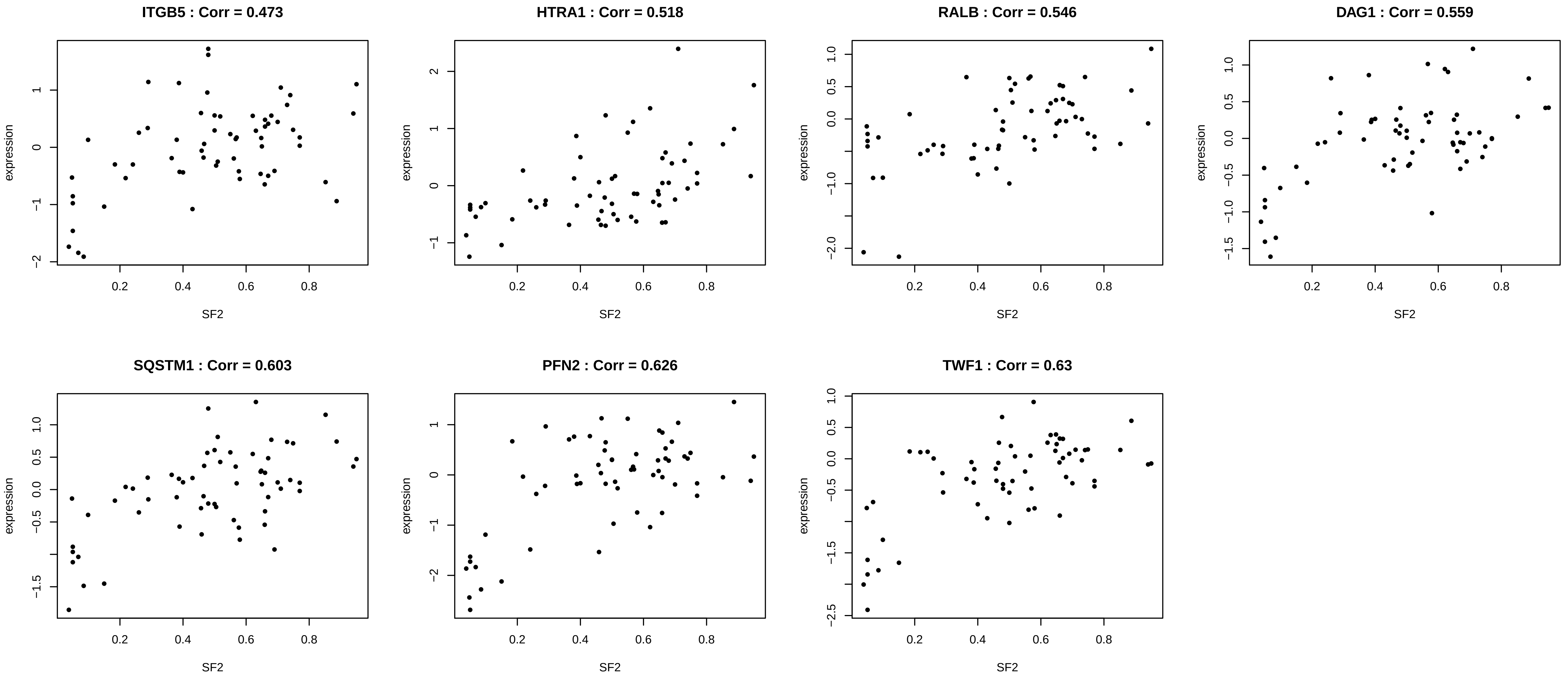
ITGB5 : Corr = 0.473
HTRA1 : Corr = 0.518
RALB : Corr = 0.546
DAG1 : Corr = 0.559
SQSTM1 : Corr = 0.603
PFN2 : Corr = 0.626
TWF1 : Corr = 0.63
expression
SF2
