# Supplementary material for: Identification of a radiosensitivity signature using integrative metaanalysis of published microarray data for NCI-60 cancer cells
Source: BMC Genomics. 2012 Jul 30;13:348. doi: 10.1186/1471-2164-13-348 (PMC3472294; doi:10.1186/1471-2164-13-348)
Supplement: Additional file 3 — Scatter plots of the 31 radiosensitivity signature genes between gene expression and radiosensitivity (SF2) in HU-6800 microarray. [file 1471-2164-13-348-S3.pdf]

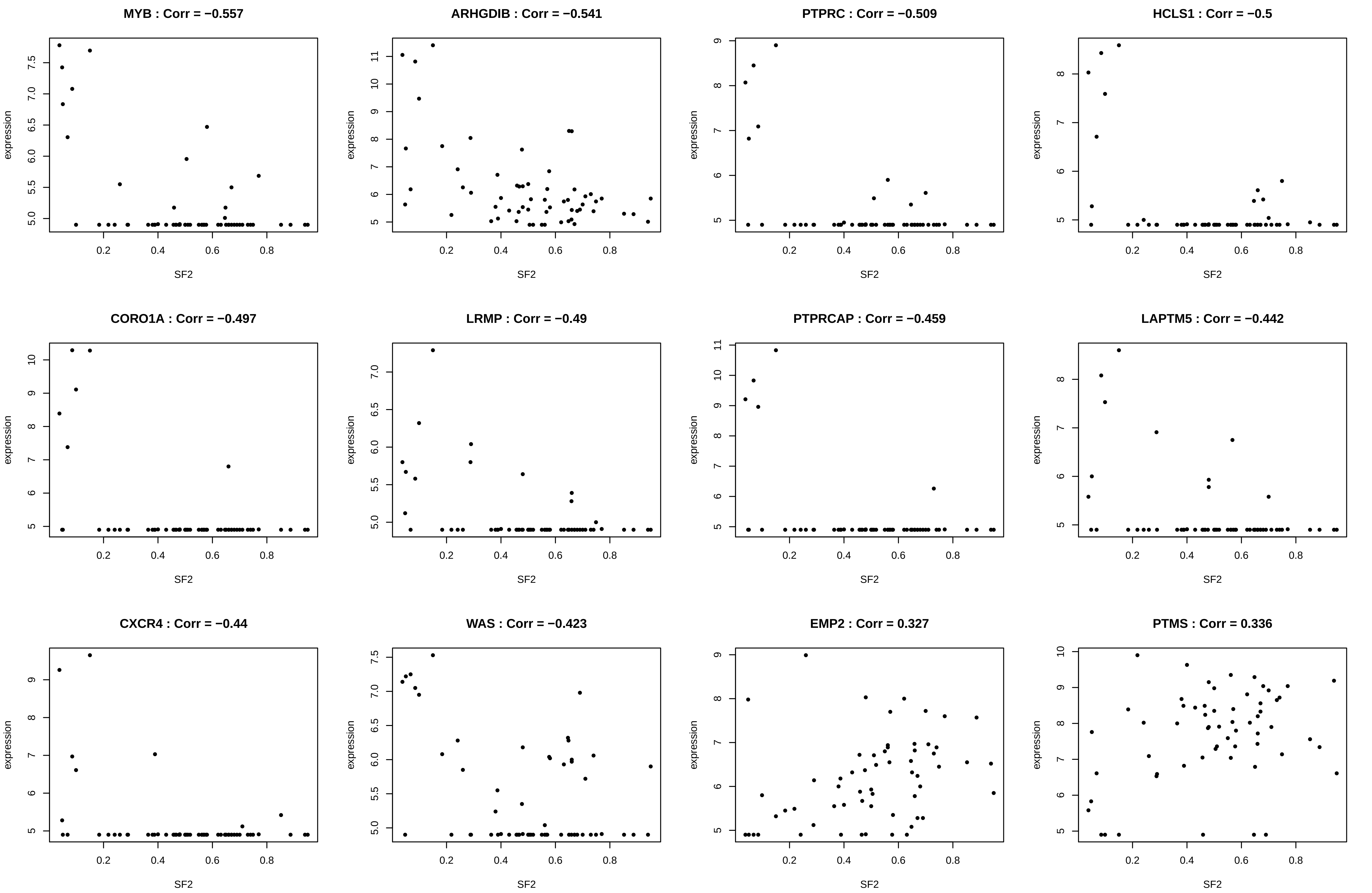
MYB : Corr = −0.557
ARHGDIB : Corr = −0.541
PTPRC : Corr = −0.509
HCLS1 : Corr = −0.5
CORO1A : Corr = −0.497
LRMP : Corr = −0.49
PTPRCAP : Corr = −0.459
LAPTM5 : Corr = −0.442
CXCR4 : Corr = −0.44
WAS : Corr = −0.423
EMP2 : Corr = 0.327
PTMS : Corr = 0.336
expression
SF2

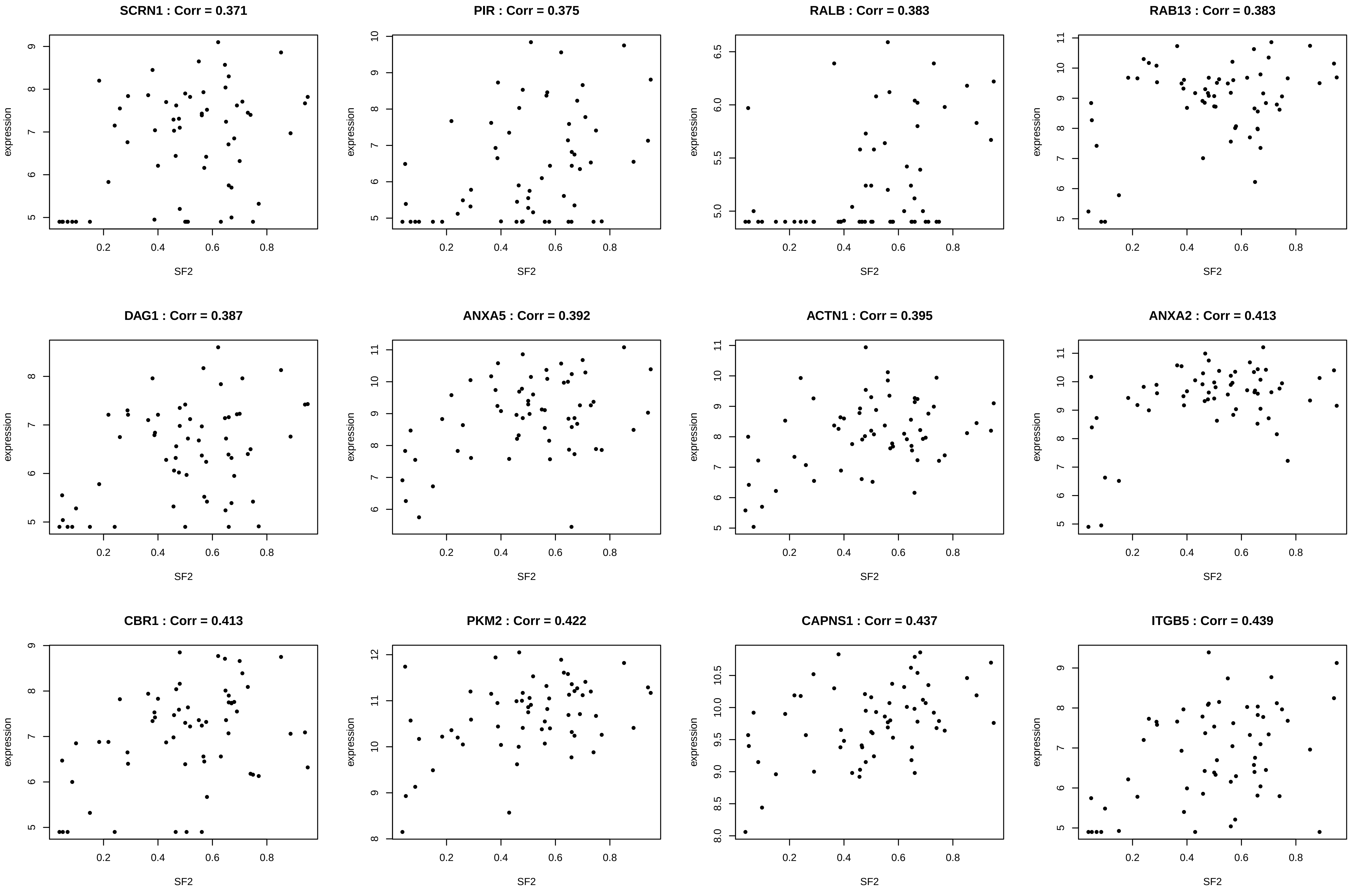
SCRN1 : Corr = 0.371
PIR : Corr = 0.375
RALB : Corr = 0.383
RAB13 : Corr = 0.383
DAG1 : Corr = 0.387
ANXA5 : Corr = 0.392
ACTN1 : Corr = 0.395
ANXA2 : Corr = 0.413
CBR1 : Corr = 0.413
PKM2 : Corr = 0.422
CAPNS1 : Corr = 0.437
ITGB5 : Corr = 0.439
expression
SF2

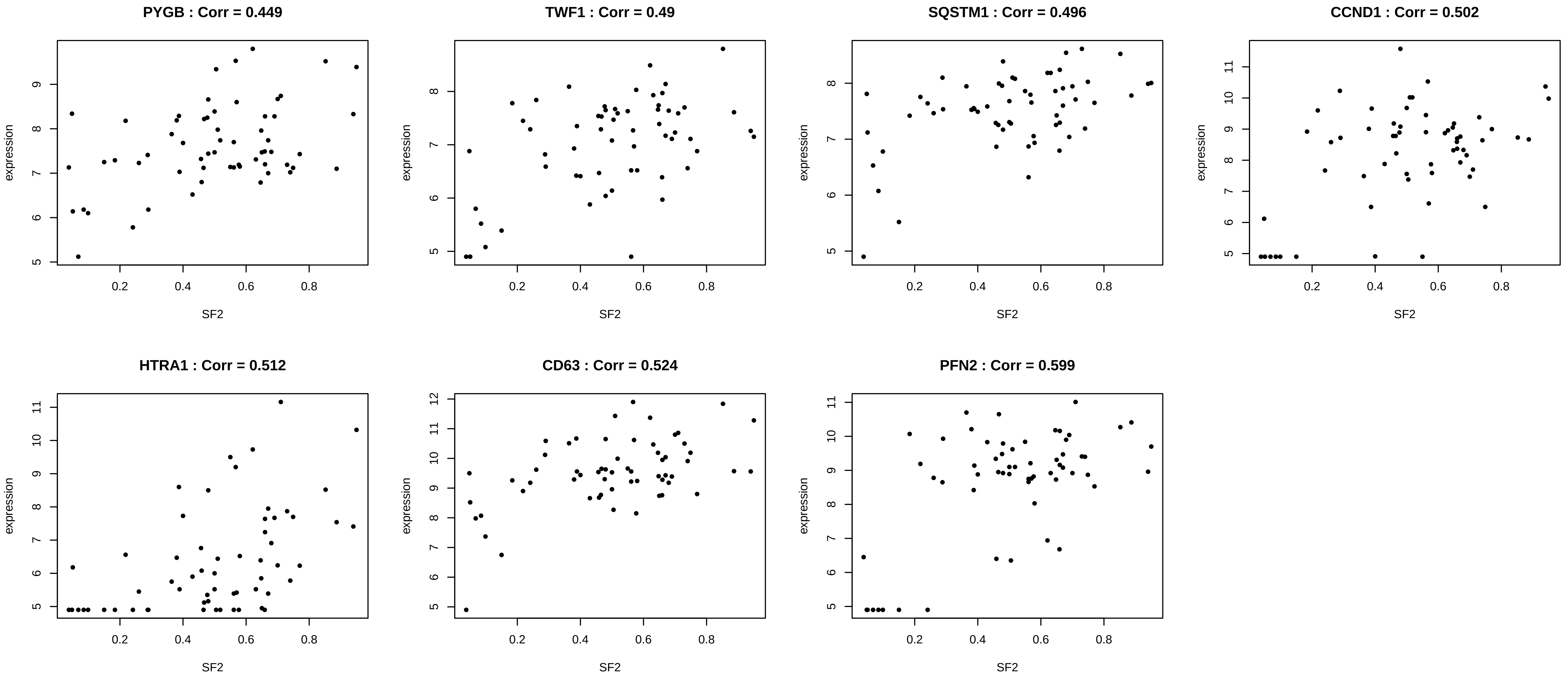
PYGB : Corr = 0.449
TWF1 : Corr = 0.49
SQSTM1 : Corr = 0.496
CCND1 : Corr = 0.502
HTRA1 : Corr = 0.512
CD63 : Corr = 0.524
PFN2 : Corr = 0.599
expression
SF2
0.2
0.4
0.6
0.8
