# Supplementary material for: Identification of a radiosensitivity signature using integrative metaanalysis of published microarray data for NCI-60 cancer cells
Source: BMC Genomics. 2012 Jul 30;13:348. doi: 10.1186/1471-2164-13-348 (PMC3472294; doi:10.1186/1471-2164-13-348)
Supplement: Additional file 4 — Scatter plots of the 31 radiosensitivity signature genes between gene expression and radiosensitivity (SF2) in Affy U133 microarray. [file 1471-2164-13-348-S4.pdf]

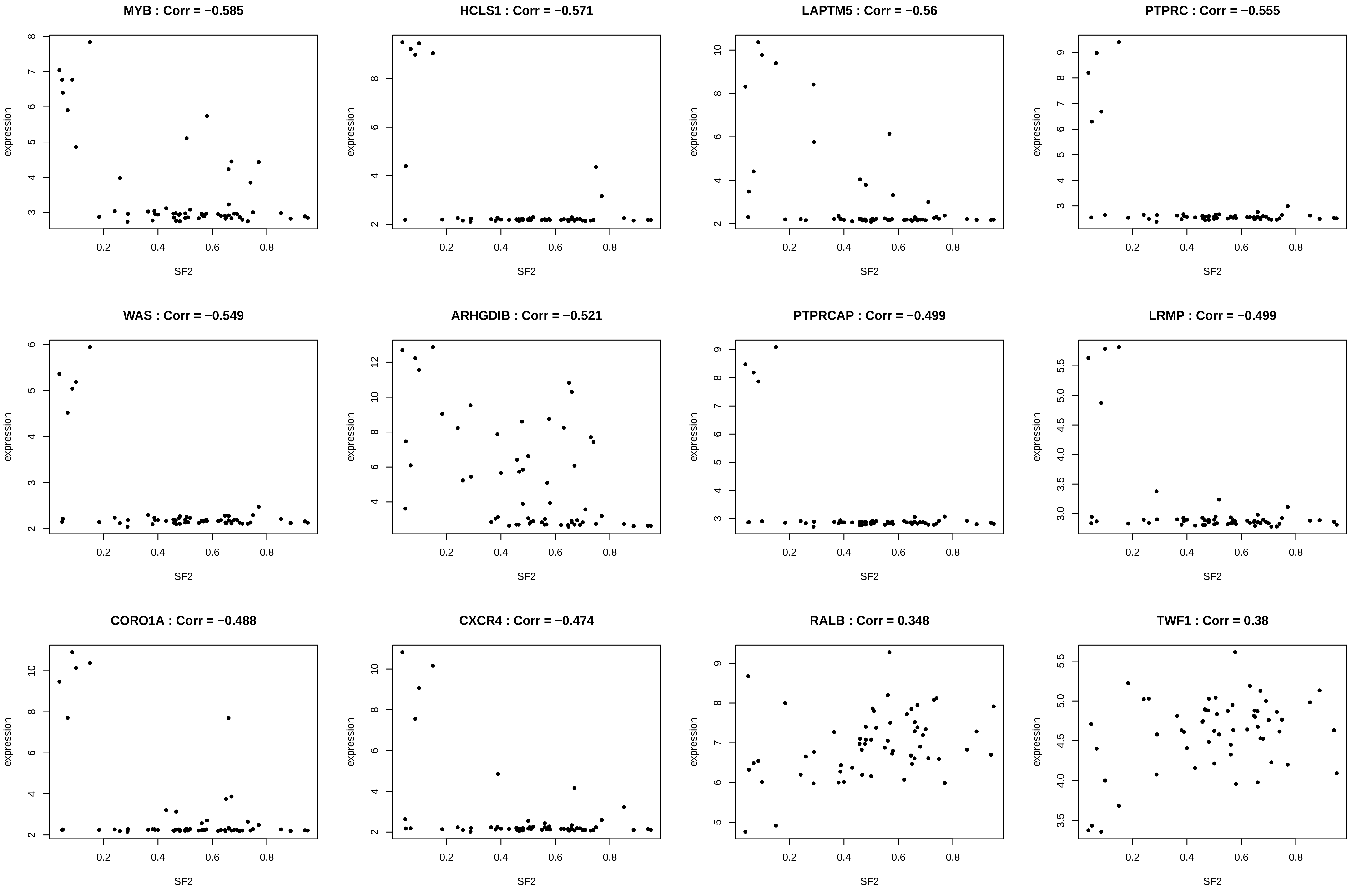
MYB : Corr = −0.585
HCLS1 : Corr = −0.571
LAPTM5 : Corr = −0.56
PTPRC : Corr = −0.555
WAS : Corr = −0.549
ARHGDIB : Corr = −0.521
PTPRCAP : Corr = −0.499
LRMP : Corr = −0.499
CORO1A : Corr = −0.488
CXCR4 : Corr = −0.474
RALB : Corr = 0.348
TWF1 : Corr = 0.38
expression
SF2

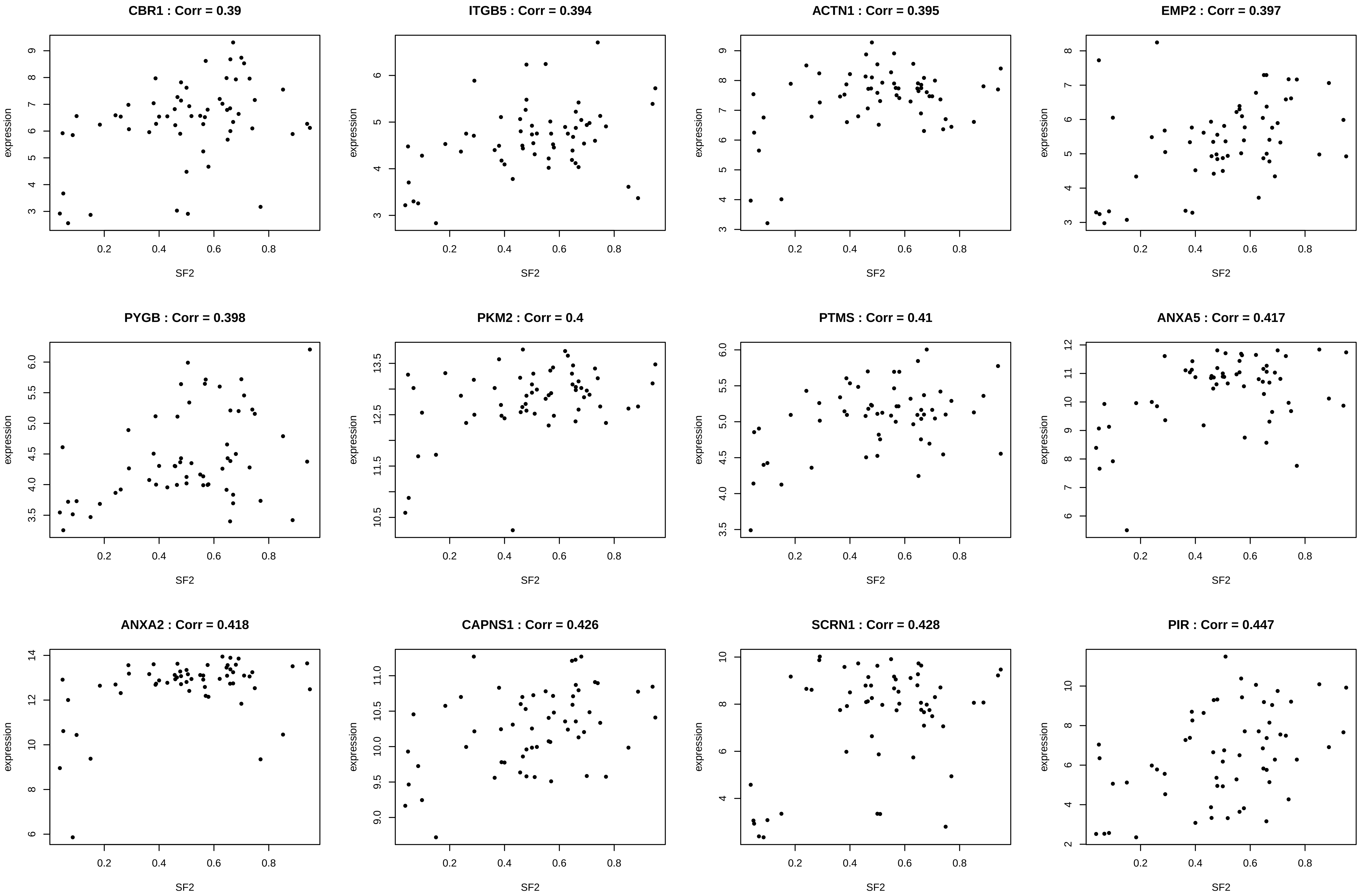
CBR1 : Corr = 0.39
ITGB5 : Corr = 0.394
ACTN1 : Corr = 0.395
EMP2 : Corr = 0.397
PYGB : Corr = 0.398
PKM2 : Corr = 0.4
PTMS : Corr = 0.41
ANXA5 : Corr = 0.417
ANXA2 : Corr = 0.418
CAPNS1 : Corr = 0.426
SCRN1 : Corr = 0.428
PIR : Corr = 0.447
expression
SF2

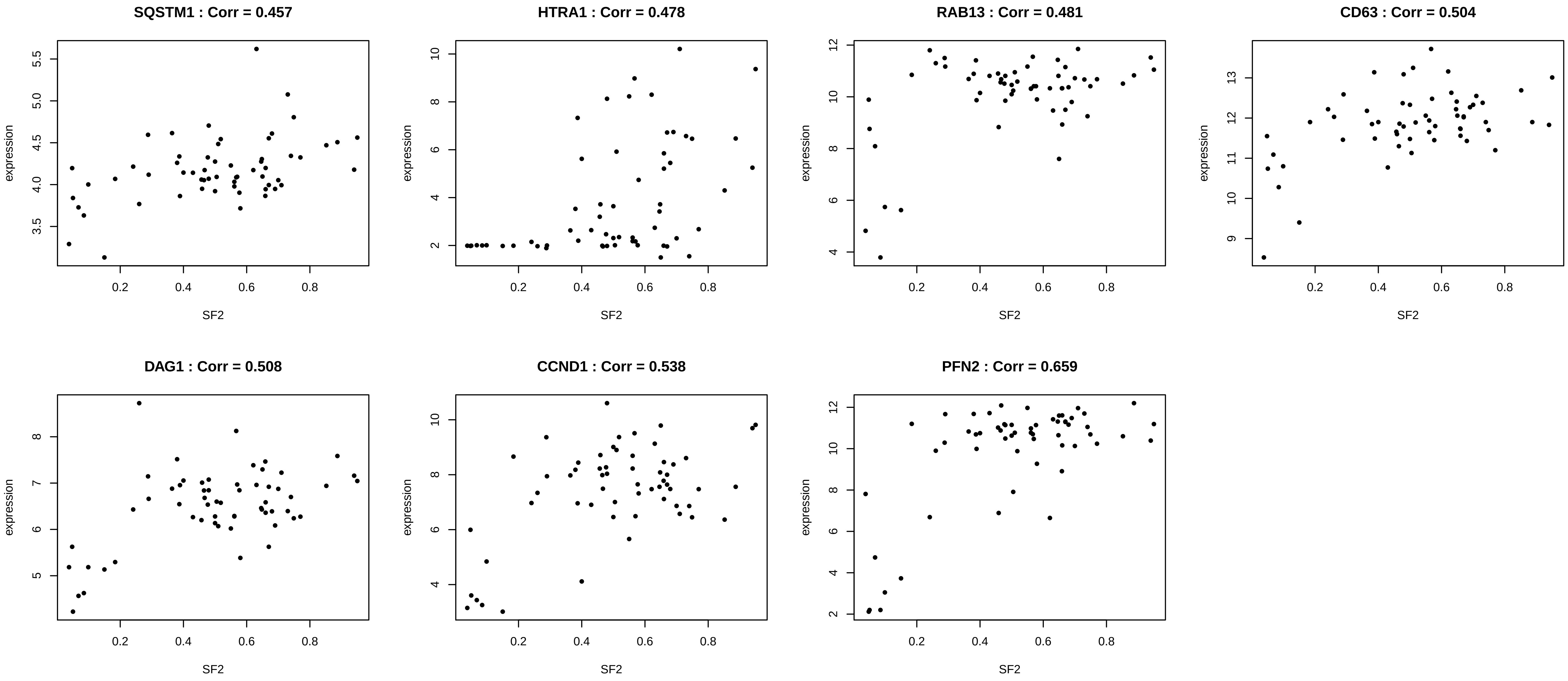
SQSTM1 : Corr = 0.457
HTRA1 : Corr = 0.478
RAB13 : Corr = 0.481
CD63 : Corr = 0.504
DAG1 : Corr = 0.508
CCND1 : Corr = 0.538
PFN2 : Corr = 0.659
expression
SF2
0.2
0.4
0.6
0.8
