# Supplementary material for: Identification of a radiosensitivity signature using integrative metaanalysis of published microarray data for NCI-60 cancer cells
Source: BMC Genomics. 2012 Jul 30;13:348. doi: 10.1186/1471-2164-13-348 (PMC3472294; doi:10.1186/1471-2164-13-348)
Supplement: Additional file 5 — Scatter plots of the 31 radiosensitivity signature between gene expression and radiosensitivity (SF2) in Affy U95 microarray. [file 1471-2164-13-348-S5.pdf]

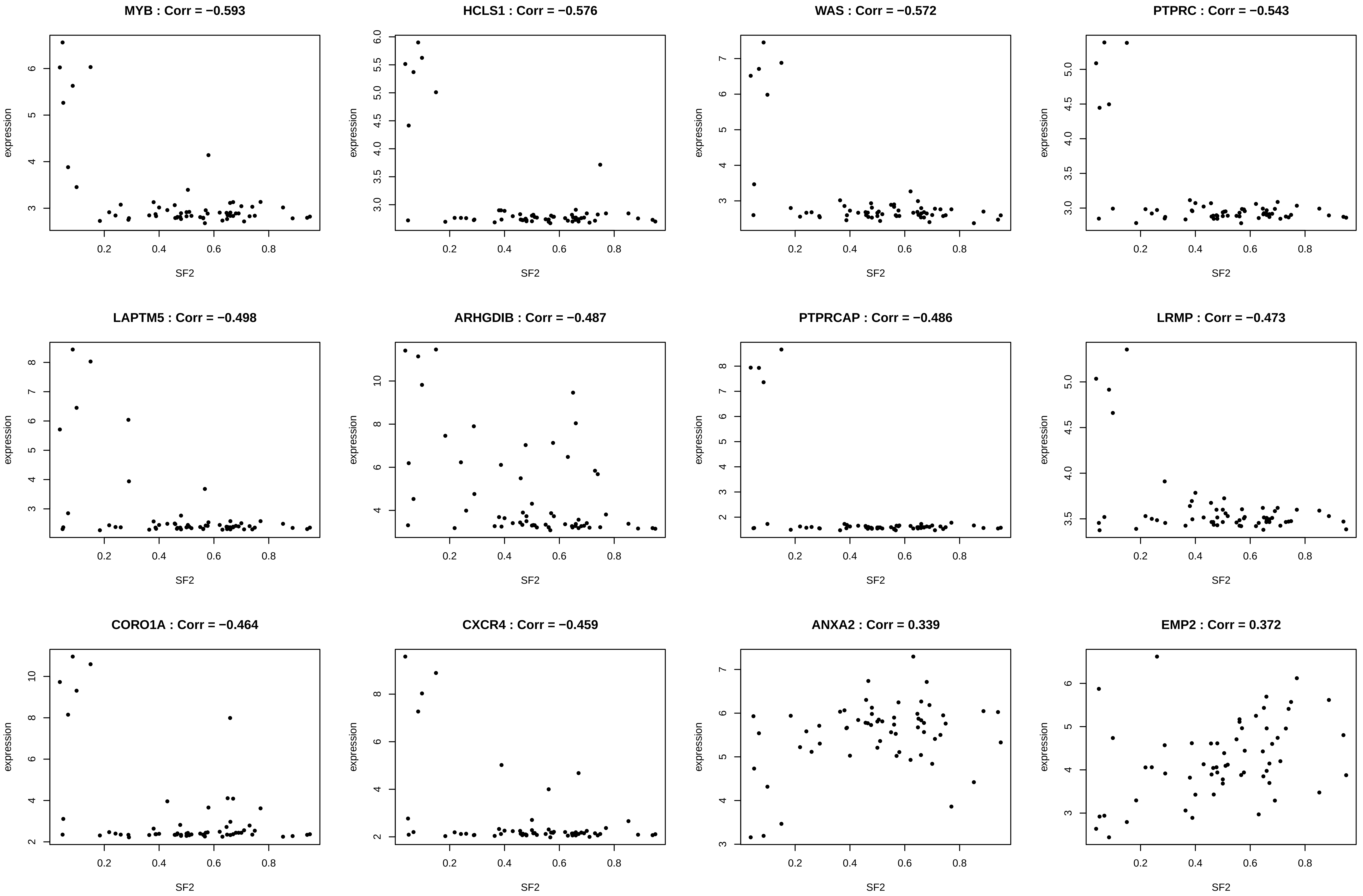
MYB : Corr = −0.593
HCLS1 : Corr = −0.576
WAS : Corr = −0.572
PTPRC : Corr = −0.543
LAPTM5 : Corr = −0.498
ARHGDIB : Corr = −0.487
PTPRCAP : Corr = −0.486
LRMP : Corr = −0.473
CORO1A : Corr = −0.464
CXCR4 : Corr = −0.459
ANXA2 : Corr = 0.339
EMP2 : Corr = 0.372
expression
SF2
0.2
0.4
0.6
0.8

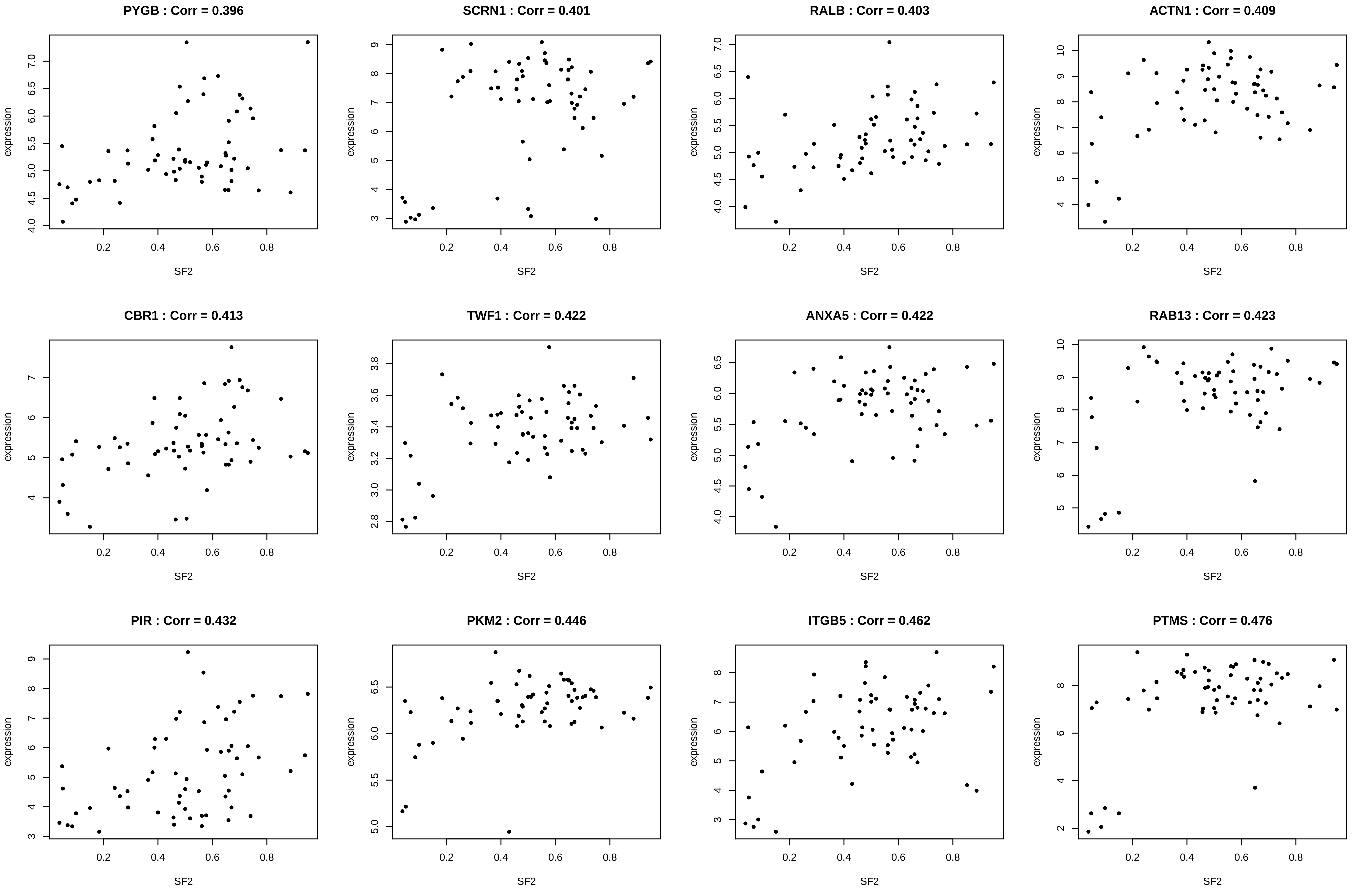
PYGB : Corr = 0.396
SCRN1 : Corr = 0.401
RALB : Corr = 0.403
ACTN1 : Corr = 0.409
CBR1 : Corr = 0.413
TWF1 : Corr = 0.422
ANXA5 : Corr = 0.422
RAB13 : Corr = 0.423
PIR : Corr = 0.432
PKM2 : Corr = 0.446
ITGB5 : Corr = 0.462
PTMS : Corr = 0.476
expression
SF2

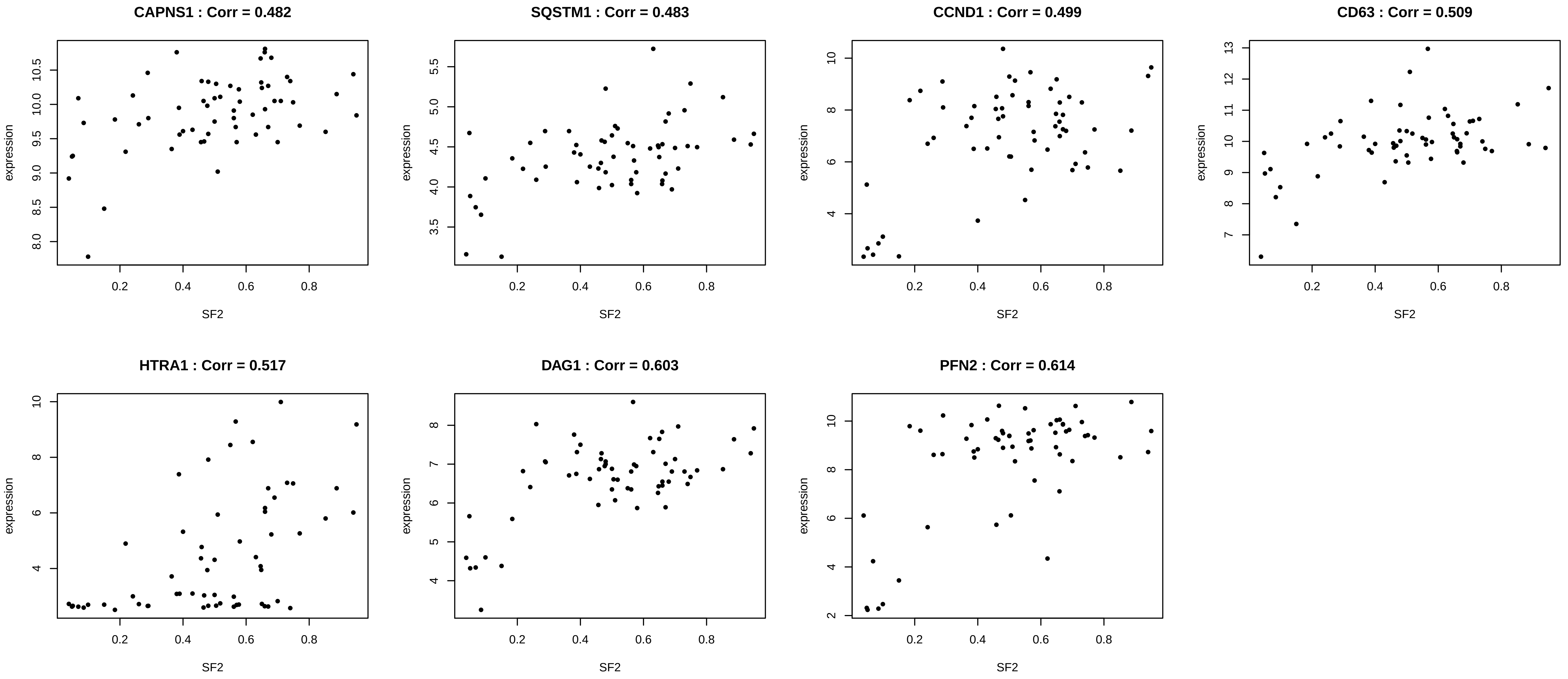
CAPNS1 : Corr = 0.482
SQSTM1 : Corr = 0.483
CCND1 : Corr = 0.499
CD63 : Corr = 0.509
HTRA1 : Corr = 0.517
DAG1 : Corr = 0.603
PFN2 : Corr = 0.614
expression
SF2
