# Supplementary material for: Identification of a radiosensitivity signature using integrative metaanalysis of published microarray data for NCI-60 cancer cells
Source: BMC Genomics. 2012 Jul 30;13:348. doi: 10.1186/1471-2164-13-348 (PMC3472294; doi:10.1186/1471-2164-13-348)
Supplement: Additional file 6 — Interaction between identified radiosensitivity genes and target genes for the integrin signaling pathway from the Ingenuity knowledge base. [file 1471-2164-13-348-S6.doc]

| Table S2. Interaction between identified radiosensitivity genes and target genes for the integrin signaling pathway from the Ingenuity knowledge base. | | |
| --- | --- | --- |
|  |  |  |
| **Identified genes in the radiosensitivity signature** | **Target genes** | **References (Pubmed IDs)** |
| PTPRC (protein tyrosine phosphatase, receptor type, C) | GRB2 (growth factor receptor-bound protein 2) | 8570203 |
|  | Fyn (FYN oncogene related to SRC, FGR, YES) | 7499298 9442882 11369760 |
|  | Src (v-src sarcoma viral oncogene homolog (avian)) | 14625311 |
| HCLS1 (hematopoietic cell-specific Lyn substrate 1) | GRB2 (growth factor receptor-bound protein 2) | 9670936 |
|  | Fyn (FYN oncogene related to SRC, FGR, YES) | 10713104 |
|  | Src (v-src sarcoma viral oncogene homolog (avian)) | 9442882 |
| RAB13 (RAB13, member RAS oncogene family) | TGFBR1 (transforming growth factor, beta receptor 1) | 15761153 |
|  | TJP1 (tight junction protein 1 (zona occludens 1)) | 8294494 |
| CCND1 (cyclin D1) | GSK3β (glycogen synthase kinase 3 beta) | 11124803 11522658 10910956 |
|  | Src (v-src sarcoma viral oncogene homolog (avian)) | 8479754 |
|  | ILK (integrin-linked kinase) | 9153256 10915780 |
| WAS (Wiskott-Aldrich syndrome (eczema-thrombocytopenia)) | GRB2 (growth factor receptor-bound protein 2) | 9307968 |
|  | Fyn (FYN oncogene related to SRC, FGR, YES) | 14707117 |
|  | Src (v-src sarcoma viral oncogene homolog (avian)) | 12769846 |
| ACTN1 (actinin, alpha 1) | Parvin-β (parvin, beta) | 15159419 |
|  | Src (v-src sarcoma viral oncogene homolog (avian)) | 16291744 |
